# Diagnosis, treatment and prevention of pediatric obesity: consensus position statement of the Italian Society for Pediatric Endocrinology and Diabetology and the Italian Society of Pediatrics

**DOI:** 10.1186/s13052-018-0525-6

**Published:** 2018-07-31

**Authors:** Giuliana Valerio, Claudio Maffeis, Giuseppe Saggese, Maria Amalia Ambruzzi, Antonio Balsamo, Simonetta Bellone, Marcello Bergamini, Sergio Bernasconi, Gianni Bona, Valeria Calcaterra, Teresa Canali, Margherita Caroli, Francesco Chiarelli, Nicola Corciulo, Antonino Crinò, Procolo Di Bonito, Violetta Di Pietrantonio, Mario Di Pietro, Anna Di Sessa, Antonella Diamanti, Mattia Doria, Danilo Fintini, Roberto Franceschi, Adriana Franzese, Marco Giussani, Graziano Grugni, Dario Iafusco, Lorenzo Iughetti, Adima Lamborghini, Maria Rosaria Licenziati, Raffaele Limauro, Giulio Maltoni, Melania Manco, Leonardo Marchesini Reggiani, Loredana Marcovecchio, Alberto Marsciani, Emanuele Miraglia del Giudice, Anita Morandi, Giuseppe Morino, Beatrice Moro, Valerio Nobili, Laura Perrone, Marina Picca, Angelo Pietrobelli, Francesco Privitera, Salvatore Purromuto, Letizia Ragusa, Roberta Ricotti, Francesca Santamaria, Chiara Sartori, Stefano Stilli, Maria Elisabeth Street, Rita Tanas, Giuliana Trifiró, Giuseppina Rosaria Umano, Andrea Vania, Elvira Verduci, Eugenio Zito

**Affiliations:** 1 Department of Movement Sciences and Wellbeing, University of Naples Parthenope, via Medina 40, 80133 Naples, Italy; 2 Pediatric Diabetes and Metabolic Disorders Unit, University of Verona, Verona, Italy; 3 Department of Pediatrics, University Hospital of Pisa, Pisa, Italy; 4 Italian Society of Pediatrics (SIP), Rome, Italy; 5 Department of Medical and Surgical Sciences, University Hospital S.Orsola-Malpighi, Bologna, Italy; 6 Department of Health Sciences, University of Piemonte Orientale, Novara, Italy; 7 Local health unit, Ferrara, Italy; 8 Italian Society for Pediatric Endocrinology and Diabetology (SIEDP), Parma, Italy; 9 Pediatrics Unit, University of Pavia and Fondazione IRCCS Policlinico San Matteo, Pavia, Italy; 10 Local health unit Roma 2, Rome, Italy; 11 Italian Society for Obesity (SIO), Francavilla Fontana (Brindisi), Italy; 12 Chair of Pediatrics, University of Chieti, Chieti, Italy; 13 Pediatric Unit, Hospital of Gallipoli, Gallipoli (Lecce), Italy; 14 Autoimmune Endocrine Diseases Unit, Bambino Gesù Children Hospital, IRCCS, Rome, Italy; 15 Department of Internal Medicine, “S. Maria delle Grazie”, Pozzuoli Hospital, Naples, Italy; 16 Department of Pediatrics, ARNAS “Civico-Di Cristina-Benfratelli”, Palermo, Italy; 17 Pediatric and Neonatal Unit, “G. Mazzini”Hospital, Teramo, Italy; 18 Department of Woman, Child and General and Specialized Surgery, University of Campania “Luigi Vanvitelli”, Naples, Italy; 19 Artificial Nutrition Unit Bambino Gesù, Children’s Hospital, IRCCS, Rome, Italy; 20 Italian Federation of Pediatricians (FIMP), Venice, Italy; 21 Endocrinology and Diabetology Unit Bambino Gesù Children Hospital, IRCCS, Rome, Italy; 22 Pediatric Unit, S. Chiara Hospital, Trento, Italy; 23 Department of Translational Medical Science, Regional Center for Pediatric Diabetes, University Federico II of Naples, Naples, Italy; 24 Azienda Tutela della Salute (ATS), Milan, Italy; 25 Division of Auxology, Istituto Auxologico Italiano, IRCCS, Verbania, Italy; 26 Pediatric Unit, University of Modena and Reggio Emilia, Modena, Italy; 27 Italian Federation of Pediatricians (FIMP), Teramo, Italy; 28 Department of Neurosurgery and Rehabilitation, AORN Santobono Pausilipon, Naples, Italy; 29 Local health unit Napoli 3 sud, Torre del Greco (Naples), Italy; 30 Research Area for Multifactorial Diseases, Children’s Hospital Bambino Gesù, Rome, Italy; 31 Istituto Ortopedico Rizzoli, Bologna, Italy; 32 Pediatric Unit, “Infermi” Hospital, Rimini, Italy; 33 Pediatric Diabetes and Metabolic Disorders Unit, University Hospital of Verona, Verona, Italy; 34 Nutrition Unit, Bambino Gesù Children’s Hospital IRCCS, Rome, Italy; 35 Local health unit (AULSS) 6 Euganea, Padova, Italy; 36 Department of Pediatrics and Infantile Neuropsychiatry, Sapienza University of Rome, Rome, Italy; 37 Hepatometabolic Unit, Bambino Gesù Children’s Hospital, IRCSS, Rome, Italy; 38 Pediatric Unit, Verona University Medical School, Verona, Italy; 39 Italian Federation of Pediatricians (FIMP), Catania, Italy; 40 Pediatric obesity Unit, ASP of Ragusa, Ragusa, Italy; 41 Oasi Research Institute –IRCCS, Troina (Ragusa), Italy; 42 Department of Obstetrics, Gynaecology and Paediatrics, Arcispedale S.Maria Nuova-IRCCS, Reggio Emilia, Italy; 43 Italian Society for Pediatric Endocrinology and Diabetology (SIEDP), Ferrara, Italy; 44 Pediatric Unit, ASST-Rhodense, Rho (Milan), Italy; 45 Deparment of Pediatrics, San Paolo Hospital, University of Milan, Milan, Italy; 46 Department of Social Sciences, University of Naples Federico II, Naples, Italy

**Keywords:** Consensus, Diagnosis, Pediatric obesity, Prevention, Treatment

## Abstract

The Italian Consensus Position Statement on Diagnosis, Treatment and Prevention of Obesity in Children and Adolescents integrates and updates the previous guidelines to deliver an evidence based approach to the disease. The following areas were reviewed: (1) obesity definition and causes of secondary obesity; (2) physical and psychosocial comorbidities; (3) treatment and care settings; (4) prevention.

The main novelties deriving from the Italian experience lie in the definition, screening of the cardiometabolic and hepatic risk factors and the endorsement of a staged approach to treatment. The evidence based efficacy of behavioral intervention versus pharmacological or surgical treatments is reported. Lastly, the prevention by promoting healthful diet, physical activity, sleep pattern, and environment is strongly recommended since the intrauterine phase.

**Electronic supplementary material:**

The online version of this article (10.1186/s13052-018-0525-6) contains supplementary material, which is available to authorized users.

## Background

Contrasting pediatric obesity is among the priority goals in the healthcare agenda of the Italian National Healthcare System. Beyond the high prevalence and persistence of pediatric obesity [1], robust evidence demonstrates that physical and psychosocial complications are already present in obese children [2] and worsen in adulthood. Therefore, prevention and treatment of pediatric obesity and complications are key strategic goals, in order to reduce morbidity, mortality, and expected costs for the care of obese adults.

The very fruitful scientific research on pediatric obesity of the last decade justified to update the guidelines, in order to provide the best evidence-based reccomendations. Therefore, the Italian Society for Pediatric Endocrinology and Diabetology and the Italian Society of Pediatrics, with other Pediatric Societies joined in the common objective of contrasting pediatric obesity, made this Consensus on “Diagnosis, therapy and prevention of obesity in children and adolescents”, updating the document published in 2006 [3].

### Methods

Four main topics were defined: 1) diagnostic criteria, secondary obesity; 2) comorbidities; 3) treatment and care settings; 4) prevention. Coordinators were identified for each topic and specific questions listed. Twenty experts’ groups were set up, embracing all the skills needed for document processing. Each group systematically revised the literature on the assigned themes limited to the time frame 1 January 2006 to 31 May 2016 and patients’ age range 0–18 years. The article search was done through PubMed using MeSH terms or descriptors. Scientific articles, systematic reviews, meta-analysis, consensus, recommendations, international and national guidelines published on pediatric obesity even prior to 2005 were considered and deemed useful to the Consensus. The level of evidence (LOE) and the grade of raccomendation were established in accordance with the National Manual of Guidelines [4] (Additional file 1). Each working group prepared a preliminary draft reporting LOE for each specific recommendation, followed by a brief description of the scientific evidence in support, epidemiological data, and any notes deemed as useful. A Consensus Conference was held in Verona, on June 9th, 2016 in the presence of the document extensors and delegates of the Scientific Societies to discuss and approve the preliminary draft. The final document was sent on October 10th, 2016 to all the extensors and members of the Pediatric Obesity Study Group of the Italian Society for Pediatric Endocrinology and Diabetology and approved on 28th February 2017 in its definitive form. Literature search was updated before preparing the final draft; no additional relevant publication was identified which might have required a change in the statements.

## Diagnosis

### Diagnostic criteria for defining overweight, obesity and severe obesity

#### The definition of overweight and obesity is based on the use of percentiles of the weight-to-length ratio or body mass index, depending on sex and age. LOE V-A

In children up to 24 months, the diagnosis of overweight and obesity is based on the weight-to-length ratio, using the World Health Organization (WHO) 2006 reference curves [5]. After the age of 2 years it is based on the Body Mass Index (BMI), using the WHO 2006 reference system [5] up to 5 years and the WHO 2007 reference system [6] thereafter (Table 1). The recommendation of using the WHO standard is based on the need to propose a reference system which, although is not an ideal model to assess adiposity in single children or groups, it has a greater sensitivity in identifying children and adolescents with overweight and obesity, in a period of particular seriousness of the pediatric obesity epidemic in Italy. On the contrary, the Italian BMI thresholds [7] underestimate the prevalence of obesity compared to WHO, probably because they were based on measurements taken during the epidemic increase of obesity [8].

**Table 1:** Diagnostic criteria to classify overweight and obesity

Age	0–2 years	2–5 years	5–18 years
Index	Weight-to-lenght ratio	BMI	BMI
Reference	WHO 2006	WHO 2006	WHO 2007
>85th percentile^a^	At risk of overweight	At risk of overweight	Overweight
>97th percentile^a^	Overweight	Overweight	Obesity
>99th percentile^a^	Obesity	Obesity	Severe obesity

^a^the 85th, 97th and 99th percentiles approximate z-scores of + 1, + 2 and + 3, respectively

#### The cut-off to define severe obesity is represented by the BMI > 99th percentile. LOE VI-B

It has been demonstrated that the 99th percentile of BMI identifies subjects with higher prevalence of cardiometabolic risk factors and persistence of severe obesity in adulthood with respect to the lower percentiles [9]. The WHO system provides the values of the 99th percentile of BMI which approximate + 3 SDS from 2 years upwards. However, as for overweight and obesity classification, the WHO terminology for severe obesity differs between younger (0–5 years) and older children/adolescents (5–18 years): the 99th percentile identifies “obesity” in the former group, and “severe obesity”in the latter. This cautious approach is motivated by the fact that the growth process differs between younger and older children; moreover few data are available on the functional significance of the cut-offs for the upper end of the BMI-for-age distribution in pre-school age [10, 11]. A scientific statement from the American Hearth Association proposed the 120% above the age and sex 95th percentile of BMI or an absolute BMI ≥ 35 kg/m^2^ (equivalent to class 2 obesity in adults) as an alternative to the 99th percentile [12]. The impact of this system using the WHO thresholds has yet to be assessed in clinical practice.

### Secondary obesity

#### The clinical suspicion of secondary obesity arises after careful anamnestic, anthropometric and clinical evaluations. LOE III-A

Obesity may be ascribed to a specific cause (endocrine, hypothalamic, genetic, iatrogenic). Therefore, clinical history, peculiar signs and symptoms must be accurately assessed such as: 1) onset of obesity before 5 years and/or rapid progression, especially in association with clues suggesting secondary causes (i.e. genetic forms); 2) continuous and/or rapid weight gain associated with reduced height velocity or short stature; 3) delayed cognitive development; 4) dismorphic features; and 5) use of drugs inducing hyperphagia (i.e. corticosteroids, sodium valproate, risperidone, phenothiazines, ciproeptadine) [13].

Early-onset obesity occurring in a child with delayed psychomotor development, cognitive deficiency, short stature, cryptorchidism or hypogonadism, dysmorphisms and characteristic facial features, ocular and/or auditory alterations, is suggestive of a syndromic form [14]. Prader-Willi syndrome is the most common one, whereas Bardet-Biedl, Alström, Cohen, Borjeson-Forssman and Carpenter are more rarely observed [15–20]. Obesity occurs frequently in children with trisomy 21, Klinefelter and Turner syndromes [21–23].

The monogenic forms, albeit uncommon, are nevertheless the most frequent causes of obesity with early onset compared to endocrine and syndromic forms [24] and are due to dysregulated hunger satiety circuits [25]. Certain monogenic forms are characterized by tall or normal stature [14]. Suspicion of syndromic or monogenic forms is confirmed by genetic investigations.

## Comorbidities

### Hypertension

#### Blood pressure measurement is recommended in all children with overweight or obesity from the age of 3 years. LOE I-A

Obesity is the main risk factor for hypertension in children and adolescents [26, 27]. The risk increases with obesity severity [28]. As blood pressure (BP) levels change according to sex, age, ethnicity and obesity, the prevalence of high BP levels and especially hypertension is heterogeneous (7–30%) in obese children [29, 30]. White coat hypertension may cause overestimation of the high BP prevalence, but the effect tends to disappear if BP is measured on at least 2–3 occasions [29].

Screening can be anticipated in children < 3 years if there is a history of neonatal complications, cardiac malformations, genetic diseases, acquired or congenital kidney diseases, neoplasms, drug use, illnesses which induce increased intra-cranial pressure [31] (**LOE III-B**).

#### The definition of high BP levels requires a precise methodology and the use of tables expressing by sex and age the percentile of systolic and diastolic blood pressure as a function of the height percentile. LOE III-A

The method of measuring BP and the definition of high systolic (SBP) and diastolic BP (DBP) values are based on the guidelines of the National High Blood Pressure Education Program Working Group on High Blood Pressure in Children and Adolescents and the European Society for Hypertension (Table 2) [32, 33].

**Table 2:** Definition of the blood pressure values

Normal BP	SBP and DBP < 90th percentile by gender, age and height
High normal BP	SBP and/or DBP ≥90th but <95th percentile by gender, age and height (BP > 120/80 mmHg even <90th percentile are considered as high normal BP).
Hypertension (Stage I)	SBP and/or DBP ≥95th <99th percentile + 5 mmHg by gender, age and height.
Hypertension (Stage II)	SBP and/or DBP ≥99th percentile + 5 mmHg by gender, age and height.

*BP* Blood pressure, *SBP* Systolic blood pressure, *DBP* Diastolic blood pressure

Primary forms of hypertension are mainly associated with obesity and more frequent in children > 6 years. Secondary forms are predominant in younger children. Nephropathy, nephrovascular pathologies and coarctation of the aorta account for 70–90% of the causes of secondary hypertension in pediatric age, while hypertension by endocrine causes is rare [34]. Various drugs (steroids, erythropoietin, theophylline, beta-stimulants, cyclosporin, tacrolimus, tricyclic antidepressants, antipsychotics, monoamino oxidase inhibitors, nasal decongestants, oral contraceptives, and androgens) can increase BP. If stage I hypertension is confirmed on 3 different visits, the following diagnostic work-up is recommended: 1) assessment of blood urea nitrogen, creatinine, glycemia, electrolytes, lipids, urine examination, microalbuminuria (may be influenced by physical activity) (**LOE II-A**); 2) measurement of glomerular filtration by formulas for renal function monitorig (**LOE III-B**); 3) echocardiography to assess organ damage (left ventricular hypertrophy, altered cardiac structure) (**LOE III-A**) [35]. Left ventricular remodeling or concentric hypertrophy are associated with high BP levels and other comorbidities such as visceral obesity and atherogenic dyslipidemia [36, 37]. Weight loss and reduced sodium intake are recommended. If stage II hypertension or secondary causes are present, the patient must be referred to a specialist for further investigations and treatment [31, 34, 35].

### Prediabetes and type 2 diabetes mellitus

#### Fasting blood glucose measurement is recommended in all children and adolescents with overweight and obesity since the age of 6, as the first step for screening prediabetes and type 2 diabetes. LOE V-A

The diagnosis of prediabetes, i.e. high fasting blood glucose and impaired glucose tolerance (IGT) or overt type 2 diabetes (T2D) is based on fasting plasma glucose or oral glucose tolerance test (OGTT) [38]. The use of hemoglobin glycosilated A1c (HbA1c) is still controversial in pediatric age [38–42]. The criteria for defining prediabetes and T2D are summarized in Table 3. The screening must be repeated after 3 years, unless rapid weight increase or the development of other cardiometabolic comorbidities occur. Since evidences provided from national studies suggest that prediabetes is already present in about 5% obese children < 10 years [43], it is recommended to start the screening by testing fasting glucose in all overweight or obese children after the age of 6 years. The OGTT is indicated after the age of 10 years or at onset of puberty in agreement with the criteria of the American Diabetes Association [38] (Table 4). Certain conditions, such as non-alcoholic fatty liver disease (NAFLD), fasting blood glucose ≥86 mg/dl, or a combination of triglycerides (TG) > 100 mg/dl plus fasting blood glucose > 80 mg/dl, or TG to HDL-cholesterol ratio (TG/HDL-C) ≥2.2, have been associated with increased risk of IGT [44–47] and therefore, an OGTT may be considered in latter cases (**LOE VI-B**) (Table 4).

**Table 3:** Criteria for the diagnosis of prediabetes and diabetes mellitus

Prediabetes Impaired fasting glucose: plasma glucose (after 8 h of fasting) between 100 (5.6 mmol/l) and 125 mg/dl (6.9 mmol/l) Impaired glucose tolerance: plasma glucose after 2 h of the OGTT between 140 and 199 mg/dl (7.8 mmol/l) HbA1c between 5.7–6.4% (39–47 mmol/mol) Type 2 diabetes Random glycemia ≥200 mg/dl (11.1 mmol/l) and symptoms suggestive of diabetes (glycosuria without ketonuria, polydipsia, weight loss). Confirmation with a second test is not necessary. If symptoms are lacking, diagnosis is made whether one of the following criteria is fullfilled: 1. Fasting glycemia ≥126 mg/dl after 8 h of fasting. 2. Glycemia ≥200 mg/dl after 2 h of the OGTT. 3. HbA1c ≥6.5% or ≥ 48 mmol/l (IFCC reference method using high-performance liquid chromatography (caution in pediatric age). If one test is positive, the diagnosis must be confirmed by a second test. Whenever the two tests are discordant, the patient should be strictly monitored and the positive test repeated within 3–6 months. If the diagnosis of diabetes is made, the assessment of the autoimmune markers (ICA, GAD, IA2, IAA o ZnT8) is needed to exclude type 1 diabetes. Genetic screening for monogenic diabetes is recommended in the rare cases presenting with obesity, diabetes, negative autoimmunity tests and family history for T2D.

**Table 4:** Indication for the oral glucose tolerance test in children and adolescents with overweight or obesity

Children with fasting plasma glucose ≥100 mg/dl or HbA1c ≥5.7–6.4% (39–46 mmol/mol) Adolescents (> 10 years of age) or at onset of puberty with overweight (BMI > 85th percentile) and at least one of the following risk factors: - Family history of T2DM in first- or second-degree relatives; - Race/ethnicity (African American, Latino, Native American, Asian American, or Pacific Islander); - Signs or conditions associated with insulin resistance (hypertension, dyslipidaemia, polycystic ovary syndrome, acanthosis nigricans, or small for gestational age at birth) - Maternal history of diabetes or gestational diabetes during the child’s gestation - Non alcholic liver disease - TG/HDL-Cholesterol ≥2.2 - Fasting plasma glucose ≥86 mg/dl - TG > 100 mg/dl and fasting plasma glucose > 80 mg/dl	

### Dyslipidemia

#### The measurement of cholesterol, HDL-cholesterol and triglycerides is recommended in all children and adolescents with obesity since the age of 6. LOE I-A

The dyslipidemic pattern associated with childhood obesity consists of a combination of elevated TG, decreased HDL-C, and low density lipoprotein cholesterol. The prevalence of dyslipidemia among obese children was 46–50.4% [48, 49]. Because the association of obesity/hyperlipidemia (expecially hypertriglyceridemia) is predictive of fatal and non fatal cardiovascular events in adult life [50], the screening of dyslipidemia is recommended, and should be repeated after 3 years, if negative, or more frequently if rapid increase in weight or development of other cardiometabolic comorbidities occurs [51, 52].

#### In the absence of national reference values, the diagnosis of dyslipidemia is based on the criteria proposed by the expert panel on integrated guidelines for cardiovascular health and risk reduction in children and adolescents. LOE III-B

The cut-offs for the definition of abnormal lipid levels as proposed by the Expert Panel [51] are summarized in Table 5. Recent studies have shown that the TG/HDL-C ratio is associated with insulin resistance and early organ damage (heart, liver, and carotid) [53–55]. The Tg/HDL-C > 2.2 can be considered as a marker of atherogenic dyslipidemia and an altered cardiometabolic risk profile in obese children in Italy [55, 56] (**LOE V-A**). Children with TG ≥ 500 mg/dL or LDL-Cholesterol persistently ≥ 160 mg/dL need lipid specialist consultation [51].

**Table 5:** References values to define dyslipidemia according to the Expert Panel on Integrated Guidelines for Cardiovascular Health and Risk Reduction in Children and Adolescents^60^

Cathegory	Acceptable	Borderline-high	High
Total cholesterol (mg/dl)	< 170	170–199	≥200
LDL-cholesterol (mg/dl)	< 110	110–129	≥130
Non HDL-cholesterol (mg/dl)	< 120	120–144	≥145
Triglycerides (mg/dl)			
0–9 years	< 75	75–99	≥100
10–19 years	< 90	90–129	≥130
	Acceptable	Borderline-low	Low
HDL-cholesterol (mg/dl)	> 45	40–45	< 40

Lipids are determined after at least 12 h of fasting

*LDL* Cholesterol is calculated by the Friedewald’s formula as total Cholesterol minus *HDL* cholesterol minus (Triglycerides/5) (provided that triglycerides are < 400 mg/dl)

*Non HDL* cholesterol is calculated as total Cholesterol minus *HDL* Cholesterol

### Gastroenterological complications

#### Non-alcoholic fatty liver disease

##### The assessment of transaminases and liver ultrasound is suggested in all children and adolescents with obesity starting at age of 6 years. LOE V-B

The prevalence of NAFLD in obese children is 38–46% [57, 58]. Bright liver on ultrasound examination, with or without elevation of alanine aminotransferase (> 26 U/L in boys and > 22 U/L in girls) suggests NAFLD [59]. Weight reduction and re-testing after 6 months are initially recommended [60] (**LOE III-A**). If liver hyperechogenicity and/or elevated alanine aminotransferase persist despite weight loss, other causes of hepatic disease (i.e. viral hepatitis, Wilson’s disease, autoimmune hepatitis, alpha 1 anti-tripsin deficiency, etc.) should be investigated. If ALT persistently exceeds twice the normal limit, the patient must be referred to a pediatric hepatologist [61].

Liver biopsy is the gold standard for diagnosis, but its invasiveness and the possible complications limit its use only to selected cases [61] (**LOE VI-A**).

Assessment of biochemical markers (i.e. retinol-binding protein 4, cytokeratine 18, hyaluronic acid) [62, 63] as indicators of hepatic histological damage, or clinical-laboratory scores as indicators of prognostic risk is not recommended in the clinical practice [64, 65] (**LOEV-D**).

Non-invasive investigations (magnetic resonance, computed tomography, elastography, ultrasound elastography) [66] are promising but again their use is not recommended. (**LOE V-D**).

NAFLD may be screened also in overweight children presenting with waist-to-height ratio > 0.5 and the assessment yearly repeated [67].

### Gallstones

#### There is no evidence to recommend the screening for colelithiasis. LOE IV-C

Gallstone disease occurs in approximately 2% obese children and adolescents [68, 69]. The rate increases up to 5.9% in obese patients with rapid weight loss [70]. The disease is rarely diagnosed, since it is symptomatic only in 20% cases [69, 71] In the presence of pain, primarily in the right upper quadrant, nausea and vomiting, assessment of serum transaminases, gamma glutamil transpherase, alkaline phosphatase, bilirubin and liver ultrasonography are diagnostic [71–73].

### Gastroesophageal reflux

#### Gastroesophageal reflux is suspected in the presence of evocative symptoms (such as pyrosis, heartburn, regurgitation). LOE VI-B

The prevalence of gastroesophageal reflux in obese children and adolescents is 13–25% (diagnosis made through questionnaires) [74–78]. Suggestive symptoms are pyrosis, epigastralgia, regurgitation. Weight loss may improve these symptoms. However, if symptoms persist or more severe symptoms occur (dysphagia, vomit) despite weight loss, referral for specialist investigations (gastrointestinal contrast study, endoscopy and oesophageal pH or impedance monitoring) and treatment is required [79].

### Polycystic ovary syndrome

#### The components of the polycystic ovary syndrome should be considered in all female adolescents with obesity. LOE VI-A

Polycystic ovary syndrome (PCOS) is characterized by hyperandrogenism (acne, hirsutism and alopecia) and ovary dysfunction (oligo-amenorrhea). It is associated with increased risk of infertility, T2D, metabolic syndrome and cardiovascular disease in adulthood [80, 81]. In adult women, the diagnosis is based on at least two of the following criteria: a) oligo-ovulation and/or anovulation; b) clinical and/or biochemical signs of hyperandrogenism; c) polycystic ovary [82]. Since there is no widely accepted definition for PCOS in the teenage, it is suggested to identify and treat the single components of the syndrome [83]. Referral for specialist investigations is required to exclude other hyperandrogenic causes (congenital adrenal hyperplasia, androgen-secreting tumors, Cushing syndrome/disease) [80–84].

### Respiratory complications

#### Respiratory symptoms and signs should be sought in children and adolescents with obesity. LOE V-A

The prevalence of respiratory problems, such as asthma, obstructive sleep apnea syndrome (OSAS), and obesity hypoventilation syndrome (OHS) is higher in obese children and adolescents compared to the general population [85, 86]. OSAS affects 13–59% of obese children [85, 87–89]. The severity is strongly associated with excess weight, while adeno-tonsillar hypertrophy, skull-facial abnormalities, Afro-American and Asian ethnicities are modulation factors [85, 90]. The OHS is less frequent, affecting 3.9% obese patients [89].

Children and adolescents may present with increased breath rate, dyspnea after moderate efforts, wheezing, chest pain. OSAS is associated with intermittent hypoxemia, hypercapnia, and disrupted sleep. Specific symptoms and signs are: snoring/noisy breathing (> 3 nights/week), pauses in breathing, mouth breathing, awakening headache that may persist during the day, daytime sleepiness, inability to concentrate, poor academic performance, hyperactivity, cognitive deficits. Rarely, growth delay, systemic hypertension pulmonary and artery hypertension have been reported in severe obesity [91, 92].

OHS is characterized by severe obesity, chronic daytime alveolar hypoventilation (defined as PaCO_2_ levels > 45 mmHg and PaO2 < 70 mmHg), a pattern of combined obstruction and restriction, in absence of other pulmonary, neuromuscular, metabolic, or chest diseases that may justify daytime hypercapnia [89].

In the presence of respiratory symptoms/signs, transcutaneous saturation of O_2_ should be determined; for values < 95%, arterial blood emogasalysis should be performed. If asthma and/or any other ventilatory dysfunction are suspected, respiratory function (spirometry, pletismography, six minute walking test) should be measured. Allergological evaluation is not necessary, unless a history of atopia is reported [86], neither is necessary measuring the exhaled nitric oxide [93, 94]. Night polysomnography is the gold standard for diagnosis of sleep disorders. The apnea/hypopnea index (ratio between total number of apnea/hypopnea episodes and duration of sleep in hours) indicates the severity (1–5 very mild; 5–10 mild; 10–20 moderate;> 20 severe). Alternatively, overnight pulse oximetry can be used, which is very specific but less sensitive. Otorhinolaryngoiatric or odontoiatric evaluations complete the diagnostic work-up. Cardiology referral should be considered in severe and long-lasting OSAS for assessing lung or systemic hypertension, and left ventricular hypertrophy [91]. Cognitive assessement may be required to assess neurocognitive damage and behavioral disorders [95].

### Orthopaedic complications

#### Orthopaedic complications should be sought in the presence of musculoskeletal pain and joint limit ation at the lower extremity. LOE V-A

Severity of obesity and sedentary lifestyle influence the morphology of osteo-cartilaginous structures and growth plate, leading to serious orthopedic consequences [96, 97]. The main orthopaedic complications are: slipped capital femoral epiphysis, Blount’s disease or tibia vara, valgus knee, flat foot [98–103].

Slipped capital femoral epiphysis may affect one or both hips; it usually occurs during the pubertal growth spurt. Hip pain and/or knee pain, an acute or insidious onset of a limp and decreased range of motion in the affected hip are the main symptoms/signs [104]. Blount disease is characterized by the varus deformity of the leg. Clinical manifestation is the instability of the knee in walking and lateral movements, simulating lameness [100]. Valgum knee is characterized by the deformity of the femoro-tibial angle in valgism; other deformities are associated, such as deviations in rotation of the tibia [101, 102]. Flat foot is characterized by flattening of the medial arch and heel valgus. Pain may be reported along the medial part of the foot, with more specific complaints after exercises or long walks [105].

#### Although obesity may exhibit higher risk of fracture, the measurement of bone density is not recommended. LOE V-D

The risk of fracture is increased in obese children, even for low energy injuries [106–108]. Inactivity, abnormalities in biomechanics of locomotion, inadequate balance may expose the obese child to fall and consequently to fracture, especially of the forearm [109]. There is no evidence that obesity results in a reduction of bone density [110]: while some studies have described an increased or normal bone mineral content, others reported a reduced bone mass in relation to bone size and weight [107].

### Renal complications

#### There is insufficient evidence to recommend screening of kidney complications in non-diabetic and non-hypertensive children and adolescents with obesity. LOE IV-D

In adults, obesity is an independent risk factor for chronic kidney disease [111]. Obesity complication, (i.e. hypertension, dyslipidemia, insulin resistance, T2D, inflammatory state, autonomous system dysfunction) indeed, can alter the kidney function [112]. Peculiar to obesity, the obesity-related glomerulopathy is a secondary form of segmental focal glomerulosclerosis occurring tipically in obese patients and that improves after weight loss [112].

Obesity is likely to be a risk factor for chronic renal disease in children too. Indeed, children with renal disease have BMI higher than healthy population [113] and kidneys transplanted from obese donors have reduced glomerular filtration and higher rate of dysfunction than the kidneys obtained from normal weight donors [114]. In the light of current evidence [115–119], the assessment of microalbuminuria is not recommended in non-diabetic and non-hypertensive obese children (**LOE IV-D**). Individual cases of severe obesity (BMI > 40) that may be associated with proteinuria in the nephrotic range remain to be evaluated individually (**LOE VI-C**).

### Idiopathic endocranic hypertension

#### Headache, vomiting, photophobia, transiently blurred vision, diplopia should be sought in subjects with overweight/obesity, especially if adolescents. LOE V-A

Idiopathic endocranial hypertension is rare but potentially serious condition that can cause permanent loss of vision [120–122]. Prevalence and risk of recurrence increase with the severity of BMI [123–125]. Some symptoms occur frequently in adolescents as in adults (headache, vomiting, photophobia, transiently blurred vision, diplopia), while irritability, apathy, drowsiness, dizziness, cervical and dorsal pain are less frequent [123, 126]. The diagnosis is based on the presence of increased intracranial pressure documented with a lumbar puncture, papilledema, normal neurologic examination results (except for cranial nerves), normal cerebrospinal fluid composition, normal appearance of neuroimaging studies, and no other identifiable cause of increased intracranial pressure [127].

### Migraine and chronic headache

#### Promoting healthy lifestyle habits and weight control can be a protective factor of migraine and chronic headache. LOE V-B

Recent studies have reported greater risk of episodic or recurrent migraine or daily chronic headache or tension headache in obese children and adolescents than the normal population [128, 129]. Some drugs used for headache and migraine have weight gain as side effect [129]. Negative lifestyle factors, which may influence the prevalence of recurrent headache, are possible targets for preventive measures [130]. An intervention study reported improvement in migraine symptoms after weight loss [131].

### Psychosocial correlates

#### Psychosocial discomfort may affect therapeutic success, therefore it should be identified as part of the multi-disciplinary assessment. LOE V-A

Recognition of psycho-social correlates (unsatisfactory body image, depressive and anxiety symptoms, loss of eating control, weight concern, dysfunctional social relationships, inactivity due to problematic body image, obesity-related stigma, low self-esteem, academic failure) is crucial to promoting specific strategies that improve the results in weight loss programs [132–134]. Although obesity is not a psychopathological and behavioral disorder, referral for specialist consult is needed in the suspicious of depressive and/or anxious symptoms, dysmorphophobic traits, suicidal risk, and eating disorders [135, 136].

### Binge eating disorder

#### The presence of binge eating disorder should be considered in the multi-professional assessment of an obese child or adolescent. LOE V-B

Binge Eating Disorder (BED) is the most common Nutrition and Eating Disorder found in pediatric obesity. It is indicative of psychopathology and is a serious risk factor for the development of obesity, especially in the presence of family history of obesity and marked negative experiences coupled with factors predisposing to psychiatric disorders [137]. BED is often preceeded by uncontrolled eating since childhood, occasional bulimia, obesity, but also by an attention deficit and hyperactivity disorder [136–138]. Upon referral to appropriate medical subspecialists and/or mental health personnel, the diagnosis of BED is critical to the therapeutic success. It may be necessary associating psychological and pharmacological therapy (only in selected cases) within the weight-loss treatment program [136, 137, 139].

## Treatment

### Changes in diet and lifestyle leading to a negative caloric balance is recommended to gradually reduce the BMI. LOE I-A

The main objective is a permanent change in the child’s eating habits and lifestyle, rather than attaining rapid weight loss through low-calorie diets. It is indispensable involving the whole family and setting realistic goals.

Further goals:

- maintaining an appropriate growth rate and achieving an healthier weight-to-height ratio;
- reducing weight excess (without necessarily achieving the ideal weight), in particular the fat mass, while preserving the lean mass;
- maintaining or promoting good mental health (self-esteem, correct attitudes toward food and body image, health related quality of life);
- treatment and improvement/resolution of complications, if present, in the shortest time possible;
- achieving and mantaining a healthier weight-to-height ratio and preventing relapses.

### Diet

#### A balanced and varied diet is recommended (LOE I-A)

The classic diet-therapy based on the prescription of a low calorie diet is not effective in the medium/long term with relapses and failures, increased risk of dropout and progression into more complicated forms [140] (**LOE III-B**).

The educational process starts from the assessment of the child’s and family’s dietary habits, by means of the assessment of meal composition, portions, frequency of food intake, food preferences or aversions, use of condiments, cooking methods and food presentation [141–145] (**LOE I-A**).

Food diary is an excellent tool for assessing eating behavior; it should be compiled by the child together with the parents or by the adolescent and evaluated by the operator [146, 147] (**LOE I-B**).

### Dietary advice

1. Eat 5 meals a day (three meals and no more than two snacks) [148] (**LOE V-B**)
2. Have an adequate breakfast [149] (**LOE II-B**)
3. Avoid eating between meals [150] (**LOE III-B**)
4. Avoid high-energy and low nutrient density foods (eg. sweetened or energizing drinks, fruit juices, fast food, high-energy snack) [151, 152] (**LOE III-B**)
5. Increase intake of fruit, vegetables and fiber rich cereals [153, 154] (**LOE VI-A**)
6. Limit portions [155, 156] (**LOE I-A**)

If a hypocaloric diet is needed, it should fulfill the National Recommended Energy and Nutrient Intake Levels, based on sex, age and ideal weight for stature (proteins 1 g/kg/day; carbohydrates 45–60% of total calories; simple sugars < 15% of total calories, lipids 20–35% of total calories starting from 4 years of age, saturated fatty acids < 10% of total calories) [157] (**LOE VI-A**).

### Efficacy of dietary regimens

There are currently no randomized controlled trials (RCTs) examining the effects of different diets on child’s weight and body composition, regardless of potential confounders such as treatment intensity, behavioral or physical activity strategies [158, 159].

#### Very low caloric diet

It is the most effective regimen in terms of weight loss [160]. One example is the protein-sparing modified fast (600–800 kcal/day, protein 1.5–2 g/kg ideal weight, carbohydrates 20–25 g/day, multivitamins + minerals, water > 2000 ml/day), which can be prescribed in selected patients with severe obesity, under close medical surveillance and in specialized pediatric centers. The aim is to induce rapid weight loss (duration of this restrictive diet no longer than 10 weeks) followed by a less restrictive diet regimen balanced in macronutrients. RCTs are not available to evaluate medium to long-term efficacy compared to other diet-therapies and possible adverse effects on growth (**LOE III-C**).

#### Traffic light and modified traffic light diets

Reduced caloric intake (1000–1500 kcal/day) is achieved trough categories of foods grouped by nutrient density [161]. They were found to produce a significant improvement of BMI in 8–12 year old children even in the long term [162] (**LOE III-C**).

#### Non-restrictive approach

It does not consider a given caloric intake or nutrient composition, rather it focuses on the consumption of low-fat and high-nutrient density foods (**LOE III-C**).

#### Replacement meals

They are not recommended, since efficacy and safety have not been tested in children/adolescents.

No significant effect has been demonstrated for diets with specific macronutrient composition and medium caloric content in children. In particular:

#### Hypocaloric diets with low glycemic index and low glycemic load

Although an effect on satiety is suggested, their superiority compared with other dietary approaches has not been proved over the medium term [163–165] (**LOE I-C**).

### Exercise

#### It is recommended to associate physical exercise to diet. LOE I-A

Physical exercise ameliorates body composition and reduces cardio-metabolic risk factors. [166–171]. Change in body composition (in particular fat reduction) rather than reduced BMI is sensitive to evaluating the effectiveness of exercise [166, 172].

It has not yet been proven which is the ideal exercise for obese children [170]. Low evidence demonstrates that combining aerobic and resistance exercises results in fat mass reduction, especially with programs of at least 2 weekly sessions and duration > 60 min [173] (**LOE I-B**).

#### The evidence is limited that exercising at higher intensity is more effective in modifying the body composition (LOE I-B).

Owing to difficulty of obese subjects to practice exercise at high intensity, there is no evidence that vigorus efforts result in greater body fat reduction [166]. Children and adolescents should practice 60 min or more of physical activity every day, which should be mainly represented by aerobic exercises at least of moderate intensity; resistance exercises are suggested for at least 3 times a week, adjusted to the physical abilities of the obese child [174, 175]. Examples of aerobic and resistance exercises for obese children and adolescents are synthetized in Table 6. The practice of recreational activities and sports that involve a large amount of body mass such as swimming, soccer, basketball, volleyball, handball, rugby or require anaerobic and neuromuscular power, such as gymnastics or judo is encouraged. In severe obesity exercises that put constant weight or repeated impact on the child’s legs, feet and hips should be avoided.

**Table 6:** Examples of aerobic and resistance exercises suggested for obese children and adolescents

Aerobic exercises^a^	exercises on treadmill, cycle ergometer, elliptical trainer water activities (swimming or water aerobics)
Resistance exercises^a^	body weight exercise (push-ups, sit-ups, abdominal crunches), lifting free weights, using weight training machines and elastic resistance bands, circuit training

^a^under qualified supervision

### Sedentary behaviors

#### It is suggested to reduce the time spent in sedentary behaviours (television viewing, videogaming, internet surfing). LOE II-B

Weight gain may be only partially due to sedentary behaviors [176–179]; in the case of television viewing, it may be associated with overfeeding [180]. Interventions targeting sedentary behaviour were more effective in children aged 5–12 years [181].

#### Use of active video games may be suggested to increase daily energy expenditure in obese and sedentary children. LOE I-B

Active video games represent an additional strategy to reduce sedentary behaviors. They do not replace ‘real’ sports activities, but can contribute to increase energy expenditure beyond the sedentary activity threshold, provided they are supervised by adults [182–187].

#### The systematic use of active video games for weight loss and improvement of body composition is not discouraged. LOE III-C

While studies are not consistent with the recommendation to use active video games to obtain weight loss or improve body composition, their use is not recommended but neither discouraged to obtain other effects (improvement in vascular response, heart rate and VO_2max_ or obesity-related comorbidities; positive psycho-behavioral and psycho-social effects) [182–184].

### Cognitive and family-based behavioral therapy

#### Cognitive behavioral treatment or family-based behavioral treatment are both recommended to favor better adhesion to diet and physical activity. Cognitive behavioral treatment LOE III- B; family-based behavioral treatment LOE I- A

Cognitive behavioral techniques are effective. Neverthless, they are not easily applicable requiring specific training of the multidisciplinary team [188–190]. The most effective techniques are goal setting, self-monitoring (through food and physical activity diaries), contingency training, stimulus control, positive reinforcement, cognitive restructuring, problem solving [191].

Family-based behavioral treatments involve multi-component interventions aimed at changing the lifestyle of the whole family, with goals shared between parents and children [191–194]. Interventions in which parents are active participants are more effective than interventions in which they are not encouraged to make their own behavioral changes. On the other hand, family-based therapies require greater investment of resources in terms of time and staff involved [188, 190, 192–198]. In children, they are more effective than treatments not involving parents. There is no robust evidence demontsrating their superiority in adolescents [189, 190, 194] (**LOE I-A**).

Therapeutic education has been proposed in the recent years, using tools of cognitive-behavioral approach and motivational interview, such as reflective listening, therapeutic alliance, family approach, modeling, motivational counseling, narrative approach, positive reinforcement, goal setting, negotiating treatment objectives. It requires professional skills of all the team members with ongoing training [199–201] (**LOE VI-B**).

Child Appetite Awareness Training and Cue Exposure Treatment are still considered experimental and require further studies [202, 203] (**LOE V-C**).

### Indicators of successful treatment

#### The BMI standard deviation score is recommended to estimate weight loss. LOE V-B

The reduction of the BMI Standard Deviation Score (BMI-SDS) is the best indicator of the weight loss amount taking into account the patient’s age and gender [204]. A reduction > 0.5, but even > 0.25 (consistent with a 1 kg/m^2^ BMI reduction or stable weight for more than 1 year in a growing child) was associated with improved body composition and decreased cardio-metabolic risk [205].

Waist circumference and waist/height ratio can be used to monitor abdominal fat variations but are subject to error and offer no benefit over BMI [204, 206–208]. The same is true for the skinfold thicknesses [204, 209].

#### Other behavioral indicators (related to diet, lifestyle, physical fitness or quality of life) can be considered if no substantial reduction in the BMI-SDS occurs. LOE VI-B

Since the percentage of weight loss is generally low, evaluation based solely on the BMI-SDS may induce a sense of failure in the family and healthcare workers. In order to maintain the adherence to treatment, a stable modification of diet, physical activity and sedentary behavior, the increase of physical fitness and improvement of the quality of life should be considered as index of good compliance [210, 211].

#### The scarce effect of treatment in the long term demands the development of long-lasting care models and their validation. LOE VI-B

The effectiveness of treatment programs based on diet and lifestyle on BMI-SDS reduction was shown only in the short term (6–12 months) [167, 212]. In a European multicentre study, the success rate (BMI SDS reduction > 0.25) was 7% at 2 years; it reached 50% in a few number of centers, which differed for the greater intensity of intervention and training of the multi-disciplinary team [213, 214]. Only two national studies based on diet education, cognitive or cognitive-behavioral strategies and family involvement reported BMI-SDS reduction of 0.44 after three years [199] and 1.49 after 5 years of follow up [188], respectively.

#### It is necessary to monitor the possible onset of eating disorders, especially when the weight loss is rapid. LOE IV-A

Dissatisfaction with body image may be related to the onset of eating disorders, especially bulimia nervosa and binge eating, but also of anorexia nervosa [215–218]. Diet education undertakings can accentuate the perceived stigma in subjects with obesity, causing drastic strategies of weight control [219, 220]. In some cases, the onset is triggered by an initially desired restriction of food, which then becomes uncontrollable. Careful evaluation of excessive weight variations and related bodily experience, especially when hypocaloric diets are prescribed, is recommended [217–219, 221].

### Pharmacological intervention

#### Pharmacological therapy can only be applied after the failure of the multidisciplinary lifestyle intervention. LOE VI-B

When clinically significant weight loss cannot be achieved through lifestyle-based interventions, use of drugs is considered, especially in severe obesity with cardiometabolic, hepatic or respiratory disorders [222–226]. Management of drugs should be done in specialist centers [225].

#### Orlistat is the only drug available for the treatment of children and adolescents with severe obesity age. LOE II-B

Few studies, with small sample size and short duration, are available on the effects of anti-obesity medications in pediatric age [227–230]. Orlistat (tetra-hydro-lipstinate) is the only drug approved for the treatment of obesity in pediatric age. It seems producing significant weight loss and favoring behavioral changes [231–233]. It does not affect the mineral balance, if the low-calorie diet is associated with normal mineral content; on the contrary, attention must be paid to prevent liposoluble vitamins deficiency [234].

### Bariatric surgery

#### Bariatric surgery is the ultimate solution in adolescents with severe obesity and resistant to all other treatments, especially when serious complications are present. LOE VI-B

The indications for surgery in the adolescent are (**LOE III-B**) [235, 236]:

- BMI ≥35 kg/m^2^ with at least one severe comorbidity, such as T2D, moderate to severe obstructive sleep apnea (AHI > 15), idiopathic endocranial hypertension, NAFLD with significant fibrosis (Ishak score > 1).
- BMI ≥40 kg/m^2^ with less serious comorbidities, such as mild sleep apnea (apnea/hypopnea index > 5), hypertension, dyslipidemia, carbohydrate intolerance.

More prudently other guidelines suggest a BMI > 40 kg/m^2^ with one severe comorbidity or > 50 kg/m^2^ with less serious comorbidities [223, 237].

Eligibility criteria are: adolescents with long lasting severe obesity; a. previous failure of any dietetic, behavioral or pharmacological intervention (after at least 12 months of intensive treatment); b. family and social support in managing the multidisciplinary care programs; c. decisional capacity for surgical management and the post-surgery follow-up; d. able to express the informed assent.

#### Surgery should be performed in a highly specialized center that guarantees the presence of an experienced multidisciplinary team. LOE III-A

The multidisciplinary team carefully evaluates the case and poses the indication for the surgey taking care of the pre-surgical assessment and post-surgical follow-up [238, 239]. The preoperative phase includes a comprehensive assessment of the patient and the family, with particular regard to physical and psychological maturation of the adolescent and his/her adherence to treatment [235, 237, 240]. Neuropsychiatric counseling should be undertaken to identify cases at risk of psychotic disorders, severe major depression, personality or eating disorders, alcoholism and drug dependence [235–237]. In the postoperative follow-up anthropometric, clinical and nutritional assessment, and counseling are performed and early or late complications are monitored.

For the adverse effect on height velocity, the adolescent should have reached adequate skeletal maturation or a pubertal stage IV according to Tanner [223, 236, 237, 241] (**LOE III-A**).

Contraindications to surgery are documented substance abuse problem and/or drug dependencies; patient inability to care for him/herself or to participate in life-long medical follow-up, no long-term family or social support that will warrant such care and follow-up; acute or chronic diseases even not directly associated with obesity threatening life in the short term; high anesthetic risk; pregnancy or planned pregnancy within the first two years after surgery, current breast-feeding [237] (**LOE VI-A**).

#### Indication for surgery must be given on a case-by-case basis by the multidisciplinary team (LOE VI-A)

Surgical procedures performed mostly by laparoscopy in adolescents and supported by at least 3 years of follow-up, are: a. restrictive interventions, including adjustable gastric bandage and sleeve gastrectomy; b. restrictive/malabsorbitive interventions, such as Roux-en Y gastric by-pass (RYGB) (**LOE III-B**).

Although the RYGB is the gold standard, there is no enough evidence to support this specific surgical technique compared to the others in terms of effectiveness, side effects, long-term complications and benefits [239]. Although studies in adolescents have increased, lack of RCTs makes it difficult to establish the effective efficacy at this age. There is no evidence or expert opinion supporting the efficacy of anticipating bariatric surgery to the teenage with respect to adults. A recent Cochrane review identified four RCTs in progress with expected results in the near future [242]. Several non-randomized and non-controlled trials were published with at least three years follow-up on the use of bariatric surgery in adolescents [243–247]. The published studies showed an average BMI decrease of 16.6 kg/m^2^ after RYGB, 11.6 kg/m^2^ after gastric bandage and 14.1 kg/m^2^ after sleeve gastrectomy [248]. All interventions have been associated with improvement or complete restoration of comorbidities. Most studies are consistent in demonstrating improvement of the quality of life [244, 248–250].

### Care settings

#### For the multifactorial nature of obesity, variability in its severity, and the health implications, treatment should be conducted in multiple settings with different levels of treatment. LOE III-A

Health services should be organized in a network of services [150, 251–254]. Fundamental is the periodic training of all network operators on motivational counseling, parenting and teamwork [251]. A child- and family-centered approach is based on sharing simple and realistic objectives about ​​eating habits, sedentary behaviours, physical activity, and verification of results related to improving nutritional status, quality of life and complications if present [255–258].

#### Primary care pediatricians represent the first level treatment. LOE III-A

Primary care pediatricians’ responsibilities are summarized in Table 7 [259, 260]. They are the reference point for obese children/adolescents and their family, participating in the various proposals for action and decisions, when a more aggressive approach is proposed (e.g., hospitalization or surgery). The efficacy of obesity treatment in the primary care setting is still modest [261, 262], but it might improve if pediatricians are assisted by other professionals experienced in pediatric obesity (dieticians/nutritionist, psychologist) and trained in family education and interdisciplinary work [258, 259, 263, 264] (**LOE VI-B**).

**Table 7:** Primary care pediatricians’ responsibilities

Conditions	Responsabilities
Risk factors: Prenatal life: first-degree familiarity for obesity, low socioeconomic status; Neonatal life: small for gestational age, or macrosomic infant; Postnatal life: no breastfeeding, early complementary feeding, excessive weight gain in the first two years of life, early adiposity rebound	Monitoring the child’s weight and length linear growth Educating to a balanced diet and healthy lifestyle since the earliest years of life Assuring appropriate timing of complementary feeding
Children and adolescents with overweight or moderate, uncomplicated obesity	Early identification of children’s excess weight Promoting parental awareness of children’s excess weight Motivating and supporting the family to change, possibly involving other professionals trained in childhood obesity
Severe obesity or psychological co-morbidity, or additional risk factors, or biochemical alterations, or treatment failure within 4–6 months	Identification of severe obesity Promoting parental awareness of children’s excess weight Motivating and supporting the family to more intensive levels of care
Suspicion of secondary obesity	Referral to specialized centers

#### District or hospital outpatient services represent the second level of care. LOE VI-A

In the second level centers, the multidisciplinary team (pediatrician, dietician and psychologist) experienced in pediatric obesity defines the clinical condition of children referred by the primary care pediatricians, and runs the multidisciplinary intervention that is centered on diet education and lifestyle modification [150, 260, 265, 266]. The patient is referred to the third level health care center in case of no response to the treatment, severe comorbidities, compromised psychological balance or significantly impaired quality of life.

#### Specialized centers for pediatric obesity represent the third level of care. LOE VI-A

Third level centers are organized on a multidisciplinary and multiprofessional basis for comorbidity management or bariatric surgery. They admit patients who are suspected of secondary obesity or require more specialistic diagnostic assessment and/or intensive care programs, including bariatric surgery. They coordinate the networking activities as well as the training of operators and promote research activities and intervention trials in the context of specific protocols [267–271].

### Transition

#### Pediatric obesity care should include a transition path from pediatric to adult care. LOE VI-B

It is necessary to test a transition model for adolescents with severe obesity and/or complications, particularly with metabolic syndrome, NAFLD, hypertension [272–274]. Unfortunately, the experience is extremely limited for the high drop-out, poor consideration about obesity as chronic illness, absence of pre-established pathways, possible transition to structures that follow the specific complications (eg. hypertension), no availability of cost-effective models [275].

## Prevention

Given the multifactorial nature of obesity, preventive interventions should be designed to modify the environmental and social determinants. Health and non-health professionals should be involved in implementing healthy food education and promoting physical activity. Promotion of balanced nutrition and healthy lifestyle implies the need to remodel economic, agricultural, industrial, environmental, socio-educational, recreational and health policies, including those aimed at contrasting socio-economic and ethnic minorities’ inequalities [276]. To be effective, actions must be multicomponent and multilevel, building agreements and alliances among many stakeholders, including families, community organizations such as schools and sport institutions, health care providers [277–279]. Primary prevention actions begin from the prenatal age, involving the “Birth Pathway” within the family counselling services, spanning to the adolescence with actions spread at individual, family and community levels [260].

### Prevention is based on behavioral modification starting from the prenatal age. LOE I-A

Lifestyle-based interventions are able to achieve mild but significant effects on dysfunctional behaviors (diet, physical activity, sedentary behaviours) and BMI [280]. Maintaining the BMI in a growing child is an important health objective. The best results have been obtained in school settings and in children 6–12 years [263]. Further studies are needed to determine the effectiveness of preventive interventions in children under 3 years and adolescents [281].

### The family involvement is strongly recommended. LOE III-A

Similarly to treatment, preventive interventions involving the whole family are recommended as more successful and long lasting compared to child-centered interventions, though they were more effective in children than adolescents [263, 282–284]. Interventions targeting at specific behaviors, such as taking fruits and vegetables and reducing sedentary behaviours have been found effective as well [283].

### Prenatal age

#### Women should start pregnancy with appropriate weight and control their weight gain following an healthy lifestyle. LOE III-A

An excessive weight gain during pregnancy is associated with fetal macrosomy and increased risk of obesity [285–290]. This effect is independent of maternal hyperglycemia, which is also a well-known risk factor for future obesity [291]. Recommended gestational weigh gain is between 11.5 and 16 Kg in normal weight women, 7 to 11.5 Kg, in overweight and 5 to 9 Kg in those who with prepregnancy obesity [292].

#### Tobacco smoke in pregnancy is banned. LOE III-A

Maternal smoking in the perinatal age increased the risk of overweight at age 7 regardless of birth weight; the risk increased for maternal smoking not only in pregnancy but also in the post-natal period. There was a dose-dependent effect. Hence, smoking exposure must be banned in pre- and post-natal life [293, 294].

## Diet

### First two years of life

#### Avoid excessive weight gain and/or increased weight-to-length ratio from the very first months of life. LOE III-A

Early rapid weight gain increases the risk of overweight and obesity in childhood [295]. Prevention in infants is focused on quality, quantity and timing of food intake. In particular:

Exclusive breastfeeding is recommended up to 6 months [296–299]. **LOE III-A.**

Solid foods and beverages other than breast milk or infant formulas should be introduced no earlier than 4 months and no later than 6 months [300–305]. **LOE III-B.**

Protein intake should be limited to less than 15% of the daily energy intake [302, 306–309]. **LOE I-B.**

Reduction of lipid intake to percentages indicated for adults is not recommended [310]. **LOE II-D**.

Sweetened drinks should be avoided [311]. **LOE III-A.**

There is insufficient evidence that complementary responsive feeding practices, such as baby-led weaning (which is associated with early satiety-responsiveness acquisition), are protective against obesity respect to usual complementary feeding mode [312–314]. **LOE V-C**.

### From preschool age to adolescence

Low energy density diet is recommended, based on the principles of the Mediterranean diet, promoting at least 5 servings of fruit and vegetables and plant based proteins [315]. Food should be distributed in no more than 5 daily meals and household consumption of meals should be promoted [316, 317]. **LOE V-A.**

The use of fast food and fast food-based venues should be limited [318, 319].**LOE V-A.**

Avoid sweetened drinks, including sports drinks and juice additives; alcoholic and energy drinks should also be avoided in adolescents [320–322]. **LOE I-A**.

## Physical activity

### It is recommended that children/adolescents spend on average 60 min a day on moderate/vigorous physical activity. LOE III-A

Prospective studies have shown a negative association between levels of physical activity and overweight/obesity [323, 324]. Even moderate physical activity is sufficient to improve aerobic fitness, an important marker of metabolic health which is independent of adiposity [325, 326]. 210, 211 Moderate physical activity is more effective and easier to implement in children who are sedentary or overweight. The increase of physical activity levels can be achieved starting from the age of 2–3 years by active play, walking, using the tricycle, and after 5–6 years, promoting also sports participation 2/3 times a week. Exercise should primarily stimulate aerobic capacity, but also strength and flexibility, be adequate to the child’s ability and stage of physical and psychomotor development [174, 175, 327].

### Sedentary behaviours

#### The use of television and electronic games is discouraged in children < 2 years of age. LOE VI-B

Although there are no specific studies on the effects of video exposure on overweight/obesity in this age group, video exposure should be discouraged since it may disturb sleep regularity [328, 329].

#### Sedentary behavior, especially the time spent in front of a screen (TV, video games, computers, mobile phones, etc.) should be reduced to less than 2 h a day in children > 2 years of age. LOE III-B

The association between sedentary behaviour, obesity and cardiometabolic risk factors is weak, and it is reduced when corrected for physical activity levels [330]. On the contrary, the evidence based on prospective studies and RCTs show a strong relationship between television hours, obesity and cardio-metabolic risk factors, presumably because overfeeding frequently occurs [331, 332].

Several studies demonstrated a greater amount of television hours in children who have a television in their bedroom, but there is no clear evidence that its removal reduces the duration of the video exposure [333]; on the contrary the installation of an electronic television time manager seems effective [334]. Decreasing sedentary behavior was more successful in reducing BMI in children 5–12 years [181]. Prospective studies showed that interrupting prolonged sedentary periods with mild physical activity had beneficial effects on metabolic outcomes in adults [335]. Although evidence is lacking in pediatric age, it is suggested breaking up prolonged sitting time at home and school.

### Sleep duration and quality

#### Adequate sleep duration and quality should be promoted in infants, children and adolescents. LOE III-B

A short sleep duration is a potential risk factor for overweight/obesity through neuroendocrine and metabolic influences [336, 337]. One meta-analysis of longitudinal studies indicated a risk of obesity more than doubled in children with a sleep duration lower than recommended [338]. Three intervention studies aimed at changing sleeping hours within a multicomponent obesity treatment were not effective in reducing the BMI [339]. Waiting for stronger evidence, we endorse the recommendation for optimal amount of sleep in children and adolescents released by the American Academy of Sleep Medicine [340] syntethized in Table 8. Turning off all “screens” 30 min before bedtime is also suggested to ensuring adequate sleep.

**Table 8:** Recommended amount of sleep in children and adolescents

4–12 months	12–16 h/day (including afternoon naps)
1–2 years	11–14 h/day (including afternoon naps)
3–5 years	10–13 h/day (including afternoon naps)
6–12 years	9–12 h/day
13–18 years	8–10 h/day

### Involvement of school settings for implementing preventive actions

#### It is recommended to include the school settings in obesity prevention programs. LOE I-A

The school is institutionally devoted to the education of children and is certainly a privileged area for the implementation of preventive actions. Studies support with moderate/high evidence that promoting healthy nutrition and physical activity at school prevent excessive weight gain and reduce the prevalence of overweight/obesity [341, 342]. The most effective and promising changes are summarized in Table 9. [334].

**Table 9:** Effective environmental strategies to prevent pediatric obesity at school

Support school personnel’s strategies for implementing health promotion programs. Improvement of overall school food environment: Removal of vending machines selling sugar sweetened beverages or snacks high in fat, sugar or salt; banning sales of this kind of food; reformulation of school lunches to reduce high calorie unhealthy food. Provision of a healthy breakfast Provision of free or low-cost fruit Provision of free/low cost water Improvement of overall school physical activity environment: Increase of the daily formal PA session organized during and after school hours. Availability of school playgrounds for structured/unstructured PA during and after regular school hours	

### Conclusions

This paper is a Consensus position document on the care of pediatric obesity in children and adolescents produced by experts belonging to the Italian Society for Pediatric Endocrinology and Diabetology and the Italian Society of Pediatrics, and endorsed by the main Italian scientific societies involved in tackling obesity and its complications.

Consistent evidences suggest that the disease-burden of obesity on the overall health starts very early in life and is particularly serious for the development of cardiometabolic disease risk factors during childhood and adolescence and the association with premature mortality in adults. Furthermore, the mechanical and psychosocial comorbidities undermine physical functioning and the health-related quality of life. Several systematic reviews and meta-analyses on treatment and prevention indicate that weight control may be obtained by multicomponent intervention focused on a life-long change in the child’s eating habits and lifestyle, involving the whole family and the surrounding social environment (school, communities). The effectiveness of treatment programs based on diet and lifestyle on excess weight reduction was shown only in the short term. Further study is needed to evaluate the effectiveness and safety of the different modalities of treatment, including pharmacotherapy and/or bariatric surgery, in the long term.

## Additional file

Additional file 1: Level of evidence and grade of recommendations according to the National Guidelines System [4]. (DOCX 15 kb)

## Abbreviations

BED: Binge eating disorder
BMI: Body mass index
BP: Blood pressure
DBP: Diastolic blood pressure
HbA1c: Hemoglobin glycosilated A1c
HDL-C: HDL-cholesterol
IGT: Impaired glucose tolerance
LOE: Level of evidence
NAFLD: Non-alcoholic fatty liver disease
OGTT: Oral glucose tolerance test
OHS: Obesity hypoventilation syndrome
OSAS: Obstructive sleep apnea syndrome
PCOS: Polycystic ovary syndrome
RCTs: Randomized controlled trials
RYGB: Roux-en Y gastric by-pass
SBP: Systolic blood pressure
SDS: Standard deviation score
T2D: Type 2 diabetes
TG: Triglycerides
WHO: World Health Organization
