# Supplementary material for: Diagnosis, treatment and prevention of pediatric obesity: consensus position statement of the Italian Society for Pediatric Endocrinology and Diabetology and the Italian Society of Pediatrics
Source: Ital J Pediatr. 2018 Jul 31;44:88. doi: 10.1186/s13052-018-0525-6 (PMC6069785; doi:10.1186/s13052-018-0525-6)
Supplement: Supplementary file 1 — Level of evidence and grade of recommendations according to the National Guidelines System [4]. (DOCX 15 kb) [file 13052_2018_525_MOESM1_ESM.docx]

Level of evidence and grade of recommendations according to the National Guidelines System [4].

| Level of evidence | | Grade of recommendations | |
| --- | --- | --- | --- |
| I | Evidence from randomized controlled studies and/or systematic reviews of randomized studies | **A** | A certain diagnostic or therapeutic intervention is strongly recommended. The recommendation is sustained by level of evidence of good quality, although not necessarily I or II |
| II | Evidence from at least one well-designed randomized controlled study | **B** | Although there are some doubts that a certain diagnostic or therapeutic intervention should always be recommended, it should be carefully considered. |
| III | Evidence from controlled trial without randomization with concurrent or historical controls or their meta-analyses. | **C** | There is significant uncertainty toward or against a certain diagnostic or therapeutic intervention. |
| IV | Evidence from non-experimental descriptive studies, including retrospective or case-control studies, and their meta-analyses. | **D** | The diagnostic or therapeutic intervention is not recommended |
| V | Evidence from se series without controls. | **E** | The diagnostic or therapeutic intervention is strongly discouraged. |
| VI | Evidence form opinions of respected authorities, or expert committees, as reported in guidelines, or consensus conferences, or opinions of the members of the working group responsible of the guidelines |  |  |
